# Association of intradialytic hypotension and convective volume in hemodiafiltration: results from a retrospective cohort study

**DOI:** 10.1186/1471-2369-13-106

**Published:** 2012-09-10

**Authors:** Franklin G Mora-Bravo, Guadalupe De-La-Cruz, Sonia Rivera, Alfonso Mariscal Ramírez, Jochen G Raimann, Héctor Pérez-Grovas

**Affiliations:** 1 Servicio de Nefrología, Hospital José Carrasco Arteaga, Cuenca, Ecuador; 2 Servicio de Nefrología, Instituto Nacional De Cardiología Ignacio Chávez, Coyacan, DF, México; 3 Renal Research Institute, New York, USA

**Keywords:** Intradialytic hypotension, Hemodiafiltration, Convective volume

## Abstract

**Background:**

Hemodiafiltration (HDF), as a convective blood purification technique, has been associated with favorable outcomes improved phosphate control, removal of middle-molecules such as Beta2-microglobulin and the occurrence of intradialytic hypotension (IDH) as compared to diffusive techniques. The aim of this retrospective cohort study in dialysis patients receiving HDF in one urban dialysis facility in Mexico City was to investigate the occurrence of IDH during HDF treatments with varying convective volume prescriptions.

**Methods:**

Subjects were stratified into equal groups of percentiles of convective volume prescription: Group 1 of 0 to 7.53 liters, group 2 of 7.54 to 14.8 liters, group 3 of 14.9 to 16.96 liters, group 4 of 16.97 to 18.9 liters, group 5 of 21 to 19.9 liters and group 6 of 21.1 to 30 liters. Logistic Regression with and without adjustment for confounding factors was used to evaluate factors associated with the occurrence of IDH.

**Results:**

2276 treatments of 154 patients were analyzed. IDH occurred during 239 HDF treatments (10.5% of all treatments). Group 1 showed 31 treatments (8.2%) with IDH whereas group 6 showed IDH in only 15 sessions (4% of all treatments). Odds Ratio of IDH for Group 6 was 0.47 (95% CI 0.25 to 0.88) as compared to Group 1 after adjustment.

**Conclusions:**

In summary the data of this retrospective cohort study shows an inverse correlation between the occurrence of IDH and convective volume prescription. Further research in prospective settings is needed to confirm these findings.

## Background

Despite continuous progress of dialysis technologies intradialytic hypotension (IDH) remains a major problem in the treatment of patients suffering from chronic kidney disease stage 5D [1-3].

Rapid removal of intravascular volume by ultrafiltration results in hemodynamic instability if it exceeds the compensatory vascular refilling from the interstitial volume [4,5]. The critical blood volume at which symptomatic hypotension occurs varies from patient to patient [4,6,7] and is influenced by myocardial contractility, heart rate, and peripheral vascular resistances [8]. It may also be noted that the vascular response to reduction in blood volume is additionally strongly determined by changes in body temperature during the treatment. Factors such as the mass transfer of sodium and calcium have also been reported to associate to hemodynamic stability.

Regular use of high-efficiency on line HDF is associated with reduced morbidity (better blood pressure control, improved lipid profile, improved anemia correction and phosphate control, improved removal of beta2-microglobulin, reduction of amyloidosis and hospitalization) [9-11]. A reduction of inflammatory markers during has also been reported recently [12]. More recently, several cohort studies have shown that high-efficiency on line HDF is associated with a reduced risk of mortality [9,13]. It has also been suggested that convective methods of blood purification may improve patient outcomes, which may include a reduction of intradialytic hypotensive episodes [14-17].

Hemodiafiltration can be categorized according to the used convective volume replacement during the course of the treatment. Low-efficiency HDF includes replacements of 5 to 14.9 liters, whereas high-efficiency HDF includes replacement of 15 to 24.9 liters. A recently published analysis of the Dialysis Outcomes and Practice Patterns Study (DOPPS) reported an association between survival and the amount of convective volume [13].

To the best of the authors’ knowledge the relation of the convective volume during HDF and intradialytic hypotension has not been studied to date. This retrospective cohort study aimed to evaluate the relation between the occurrence of intradialytic hypotension and the use of different amounts of convection volume during HDF.

## Methods

### Patients selection

The database contains records of 167 patients and 6137 treatments. We selected cases for analysis from that group.

Subjects received thrice-weekly HDF sessions for a minimum duration of 3 hours during the period from 07/01/2005 and 07/16/2006 in the dialysis unit of the Instituto Nacional De Cardiología Ignacio Chavez in Mexico City, were included in this analysis. Patients were stratified into 6 equal groups of convection volume prescription (based on percentiles of convective volume in the entire dataset), and for a confirmatory analysis into 7 groups, where the reference group was not receiving HDF with convective volume. This retrospective study was approved by the Institutional Review Board of Instituto Nacional de Cardiología Ignacio Chávez (Reference #: IMIN-2010-TL-27).

### Treatment characteristics

Extracorporeal blood Flow (Qb) was chosen according to the unit's policy and was aimed keep the arterial pressure between −200 to −250 mmHg, but with a prescription limit of 500 ml/min. Patients received systemic anticoagulation with heparin-sodium 2.000 units at the beginning of treatment and 1000 units per hour. In addition dialysis filters and -lines were also heparinized with 1000 units of sodium-heparin in a 1 liter of 0.9% saline solution.

Postdilutional HDF sessions were delivered by Fresenius 4008 H dialysis machines (Fresenius Medical Care Germany, Bad Homburg, Germany). The machines are equipped with a pre pump measuring system for dynamic arterial line pressure, blood temperature monitor (BTM) for measurements of access recirculation, an on-line clearance monitor (OCM) and the blood Volume Monitor (BVM). Urea Kinetic Volume in Litters was determined by Watson Formula [18].

High-Flux polysulfone membrane dialyzers F-60 and F-80 were used (Fresenius Medical Care North America, Walnut Creek, CA, USA). Dialysis filters and blood lines were re-used up to 10 ± 4 times and disinfected by formaldehyde after each treatment. Dialysate/infusate compositions were sodium 135 mEq/L, potassium 3 mEq/L, calcium 3 mEq/L, bicarbonate 30 mEq/L and glucose 200 mg/dL. Ultra pure bicarbonate dialysate was delivered. The studied patients had no dietary fluid or salt restrictions, but were recommended high protein and calorie intake. Prescription of sufficient convective volume was made aiming for a trans-membranous pressure of 200 mmHg.

IDH was defined as per the K/DOQI guidelines [19], defined as a decrease in systolic BP of 20 mmHg or a decrease in mean arterial pressure (MAP) by 10 mmHg associated with clinical events, such as headache, nausea, vomiting and cramps, and the need for nursing interventions as per standard of care (injection of a 50 mL bolus of hyperosmolar glucose, placing the patient in Trendelenburg position and temporary cease of ultrafiltration).

#### UF rate algorithm

The relative blood volume (RBV) was measured on line with the Blood Volume Monitor (BVM). At the start of hemodialysis the RBV was equal to 100%. We program the critical limit for stopping ultrafiltration, when RBV is less than 78%. We program the ultrafiltration maximum rate with a value of 3000 mL/hour.

### Statistical analysis

Data was tested for normal distribution by Shapiro-Wilk test. Parametric data was reported as mean standard deviation and non-parametric as median [Inter quartile Range (IQR) from 25th to 75th percentile]. Differences between groups were tested by means of Analysis of Variance (ANOVA) and Post-Hoc Bonferroni-Dunnett Test. Logistic Regression analysis with only groups of convective volume included was employed to test the hypothesis that convective volume associates to the occurrence of IDH. Additional adjustment of the model by relevant clinical parameters [pre HDF systolic blood pressure (SBP), albumin, ultrafiltration rate, pre HDF body temperature, post HDF weight] was employed to exclude confounding of the results.

## Results

### Study cohort

Records of 2276 post-dilution HDF session of 154 patients were included in this retrospective cohort study. Patients were categorized in six equal groups according to percentiles of convective volume prescription data: Group 1: 0 to 7.53 liters, Group 2: 7.54 to 14.8 liters; Group 3: 14.9 to16.96 liters; Group 4: 116.97 to 18.9 liters; Group 5: 19.1 to 21 liters, and Group 6: 21.1 to 30 liters. All patients were of Hispanic ethnicity, the rest of the demographics of the studied cohort are shown in Table 1. None of the subjects had an active prescription of antihypertensive medication.

**Table 1:** Patients demographics

**Parameter**	**Sixtiles of convective volume prescription**						**Anova**	**Dunnett Post Hoc Test**	
	**Group 1**	**Group 2**	**Group 3**	**Group 4**	**Group 5**	**Group 6**		**P<0.05**	**P>0.05**
Patients [count]	23	25	22	29	27	28			
Treatments [count]	379	382	377	382	380	376			
Age [Years]	43.4 ± 15	44.5 ± 16	48.2 ± 11	43.2 ± 16	46.3 ± 14	48.9 ± 12	NS	None	1 vs. 2-6
Gender Female [count [%]]	11 [47.8%]	12 [48%]	10 [45.5%]	14 [48%]	12 [44%]	11 [39%]	NS	Chi Square Test	
Pre HDF Weight [Kg]	63.8 ±13.0	61.6 ±12.5	61.4 ±13.0	61.0 ±12.8	60.5 ±12.7	60.8 ±15.3	NS	None	1 vs. 2-6
Post HDF Weight [Kg]	61.4 ± 13.0	58.5 ± 12.2	58.7 ± 13.1	58.2 ± 12.8	57.9 ± 12.6	59.4 ± 13.0	NS	None	1 vs. 2-6
Temperature pre HDF [Celsius]	36.72 ± 0.36	36.55 ± 0.45	36.56 ± 0.42	36.54 ± 0.41	36.55 ± 0.43	36.55 ± 0.42	NS	None	1 vs. 2-6
Temperature post HDF [Celsius]	36.98 ± 0.39	36.81 ± 0.50	36.84 ± 0.48	36.85 ± 0.39	36.85 ± 0.39	36.82 ± 0.38	NS	None	1 vs. 2-6
Delta Temperature [Celsius]	0.27 ± 0.29	0.27 ± 0.38	0.28 ± 0.37	0.30 ± 0.29	0.28 ± 0.27	0.28 ± 0.28	NS	None	1 vs. 2-6
Interdialytic Weight Gain [% post HDF target weight]	4.2 ± 2.0	5.2 ± 2.1	4.8 ± 2.5	5.0 ± 2.3	4.6 ± 2.2	4.0 ± 2.1	<0.0001	1 vs. 2-4	1 vs. 5-6
Pre HDF Systolic Blood Pressure [mmHg]	136 ± 29	147 ± 28	143 ± 28	139 ± 27	137 ± 26	136 ± 26	<0.0001	1 vs. 2-3	1 vs. 4-6
Pre HDF Diastolic Blood Pressure [mmHg]	70 ± 20	77 ± 21	75 ± 22	75 ± 23	71 ± 22	69 ± 21	<0.0001	1 vs. 2-4	1 vs. 5,6
Pre HDF MAP Blood Pressure [mmHg]	93 ± 23	102 ± 23	99 ± 23	98 ± 23	95 ± 22	93 ± 22	<0.0001	1 vs. 2-4	1 vs5,6
Pre HDF Pulse Rate [bpm]	94 ± 15	93 ± 16	94 ± 18	95 ± 15	94 ± 16	94 ± 16	NS	None	1 vs.2-6

Group 1: 0 to 7.53 liters, Group 2: 7.54 to 14.8 liters; Group 3: 14.9 to 16.96 liters; Group 4: 16.97 to 18.9 liters; Group 5: 19.1 to 21 liters, and Group 6: 21.1 to 30 liters. Not significant (NS).

### Treatment characteristics

HD parameters are shown in Table 2. Treatment time, conductivity, dialysate temperature, arterial and venous line pressure, sodium prescription was similar in all studied groups (Table 2). Small significant differences between the groups were found for processed blood volume, total spent dialysate, blood and dialysate flow (Table 3). The achieved ionic Kt/V in the studied treatments showed significant differences between the groups, with higher values associating to higher convection volumes. Information on interdialytic weight gain expressed as percentage of dry weight is in the Table 1.

**Table 2:** Characteristics of hemodiafiltration (HDF) treatments

**Parameter**	**Group 1**	**Group 2**	**Group 3**	**Group 4**	**Group 5**	**Group 6**	**Anova**	**P<0.05**	**P>0.05**
Effective Dialysis Time [min]	217 ± 14	205 ± 17	210 ± 18	214 ± 17	217 ± 17	219 ± 16	NS	None	1 vs. 2-6
Total Blood Volume [liters]*	88.6 ± 14.5	74.8 ± 15.8	82.5 ± 13.4	88.7 ± 12.6	92.2 ± 11.4	97.2 ± 10.5	<0.0001	1 vs.5,6.	1 vs.2-4
Extracorporeal Blood Flow [mL/min]	406 ± 63	361 ± 71	389 ± 62	412 ± 58	423 ± 55	442 ± 52	<0.0001	1 vs.5,6.	1 vs.2-4
Dialysate Flow [ml/min]	522 ± 41	613 ± 36	632 ± 37	643 ± 45	672 ± 47	703 ± 34	<0.0001	1 vs.2-6	None
Ultrafiltration [litters]	2.62 ± 0.85	3.05 ± 1.03	2.89 ± 1.12	2.97 ± 1.14	2.67 ± 1.09	2.41 ± 1.04	<0.0001	1 vs.2-4	1 vs.5,6
Conductivity [mS/cm^3^]	14.0 ± 0.2	14.0 ± 0.1	14.1 ± 0.1	14.1 ± 0.1	14.1 ± 0.1	14.1 ± 0.1	NS	None	1 vs.2-6
Dialysate temperature [Celsius]	35.74 ± 0.35	35.64 ± 0.37	35.64 ± 0.38	35.62 ± 0.35	35.64 ± 0.30	35.68 ± 0.32	NS	None	1 vs.2-6
Arterial Line Pressure [mmHg]	−201 ± 35	−205 ± 34	−211 ± 29	−218 ± 26	−217 ± 27	−215 ± 28	NS	None	1 vs.2-6
Venous Line Pressure [mmHg]	205 ± 53	176 ± 59	176 ± 46	180 ± 41	186 ± 40	198 ± 44	NS	None	1 vs.2-6
Dialysate sodium prescription [mEq/L]	138 ± 0.7	138 ± 0.8	138 ± 0.6	138 ± 0.6	138 ± 0.6	138 ± 0.4	NS	None	1 vs.2-6

* Total Blood Volume = Qb*Dialysis Time. Group 1: 0 to 7.53 liters, Group 2: 7.54 to 14.8 liters; Group 3: 14.9 to 16.96 liters; Group 4: 16.97 to 18.9 liters; Group 5: 19.1 to 21 liters, and Group 6: 21.1 to 30 liters. Not significant (NS).

**Table 3:** Clinical parameters

**Parameter**	**Group 1**	**Group 2**	**Group 3**	**Group 4**	**Group 5**	**Group 6**	**Anova**	**P<0.05**	**P>0.05**
**Recirculation fraction [%]**	13.0 ± 6.7	14.4 ± 9.0	12.1 ± 6.6	12.5 ± 8.7	13.8 ± 8.5	13.1 ± 7.4	NS	None	1 vs.2-6
**Ionic Kt/V**	1.37 ± 0.33	1.27 ± 0.34	1.39 ± 0.36	1.44 ± 0.41	1.60 ± 0.39	1.66 ± 0.38	<0.0001	1 vs.5,6	1 vs.2-4
**Urea Kinetic Volume [Liters]**	31.3 ± 5.5	31.0 ± 6.8	33.2 ± 7.3	33.0 ± 7.0	31.7 ± 7.3	33.7 ± 7.9	NS	None	1 vs.2-6
**Clearance [ml/min]**	183 ± 36	172 ± 50	196 ± 36	209 ± 32	219 ± 31	235 ± 31	<0.0001	1 vs.3-6	1 vs.2
**Minimum RBV [%]**	82.5 ± 7.3	80.1 ± 7.2	80.3 ± 8.6	80.3 ± 8.3	82.4 ± 8.5	85.2 ± 7.4	<0.001	2 vs.6	1 vs .2-6
**Haemoglobin post HDF [g/dL]**	9.4 ± 2.7	10.1 ± 2.6	9.8 ± 2.8	9.7 ± 2.9	9.0 ± 2.7	8.4 ± 2.4	<0.0001	1 vs.2	1 vs.3-6
**Haemoglobin pre HDF [g/dL]**	7.2 ± 1.8	7.5 ± 1.9	7.2 ± 1.8	7.2 ± 2.0	6.8 ± 1.8	6.7 ± 1.8	<0.0001	2 vs.6	1 vs.2-6
**pre HDF BWC [%]**	86.4 ± 1.4	86.1 ± 1.6	86.4 ± 1.6	86.4 ± 1.8	86.7 ± 1.7	86.8 ± 1.6	NS	None	1 vs.2-6
**post HDF BWC [%]**	84.5 ± 2.3	83.9 ± 2.3	84.2 ± 2.4	84.3 ± 2.5	85.0 ± 2.1	85.4 ± 2.1	<0.0001	1 vs.5-6	1 vs.2-4
Δ **Haemoglobin [g/dL]**	2.2 ± 1.3	2.7 ± 1.4	2.6 ± 1.5	2.5 ± 1.4	2.2 ± 1.3	1.8 ± 1.0	<0.0001	1 vs.2-4	1 vs.5,6
Δ **BWC [g/dL]**	1.9 ± 1.1	2.2 ± 1.2	2.2 ± 1.3	2.1 ± 1.2	1.8 ± 1.1	1.4 ± 0.9	<0.0001	1 vs.2-4	1 vs.5,6.

Hemodiafiltration (HDF). Blood Water Component (BWC). Δ Hemoglobin = pre-post HDF, Δ BWC = pre – post HDF. Group 1: 0 to 7.53 liters, Group 2: 7.54 to 14.8 liters; Group 3: 14.9 to 16.96 liters; Group 4: 16.97 to 18.9 liters; Group 5: 19.1 to 21 liters, and Group 6: 21.1 to 30 liters.

### Intradialytic hypotension

**Figure 1:**
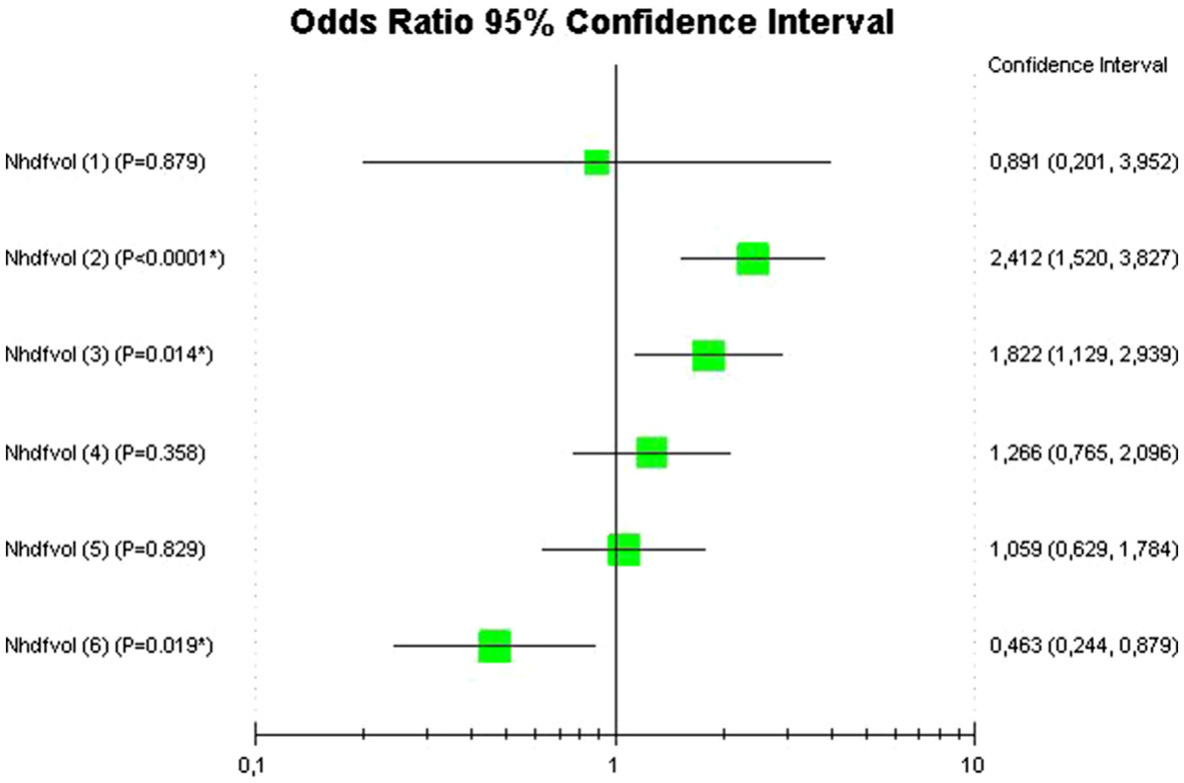
**Forest plot diagram to represent odds ratio of hemodiafiltration volume for Intradialytic Hypotension.** The groups of convective volume were defined as follows: Group 1: 0 to 0.2 liters. Group 2: 0.3 to 7.53 liters, Group 3: 7.54 to 14.8 liters; Group 4: 14.9 to 16.96 liters; Group 5: 16.97 to 18.9 liters; Group 6: 19.1 to 21 liters, and Group 7: 21.1 to 30 liters.

During the study period, IDH occurred in 239 study treatments (10.5% of all studied HD sessions; Table 4). Logistic regression was employed to analyze the association of convective volume to the occurrence of intradialytic symptoms. The first model included only groups of convective volume (Table 5a)and showed a significantly decreased risk of IDH as compared to the reference group. After inclusion of relevant clinical parameters this relation remained significant (Table 5b). For the confirmatory analysis all subjects were stratified in 7 groups of convective volume, where the reference group did receive HDF with convective volume. The results were similar to the primary analysis and showed that Group 7 had a lower risk of IDH as compared to group 1 and 2 (Table 6 and Figure 1). Ultrafiltration rate register each 15 minutes is in the Table 7.

**Table 4:** Occurrence of intradialytic hypotension (IDH) during hemodiafiltration (HDF) treatments stratified into sixtiles according to the convective volume used

	**Sixtiles of convective volume prescription**						**Total**
	**Group 1**	**Group 2**	**Group 3**	**Group 4**	**Group 5**	**Group 6**	
**Treatments [count]**	379	382	377	382	380	376	2276
**no IDH [Count [%]]**	348 [91.8%]	314 [82.2%]	324 [85.9%]	343 [89.8%]	347 [91.3%]	361 [96.0%]	2037 [89.5%]
**IDH [Count [%]]**	31 [8.2%]	68 [17.8%]	53 [14.1%]	39 [10.2%]	33 [8.7%]	15 [4.0%]	239 [10.5%]

Group 1: 0 to 7.53 liters, Group 2: 7.54 to 14.8 liters; Group 3: 14.9 to 16.96 liters; Group 4: 16.97 to 18.9 liters; Group 5: 19.1 to 21 liters, and Group 6: 21.1 to 30 liters.

**Table 5:** Logistic regression analysis to determine odds ratio of hemodiafiltration volume for intradialytic hypotension (IDH)

**a]**	**B**	**S.E.**	**Wald**	**df**	**Sig.**	**Exp[B]**	**95,0% C.I. for EXP[B]**	
							**Lower**	**Upper**
**Groups**			43.31	5	<0.0001	1		
**Sixtil (1)**	0.888	0.230	14.88	1	<0.0001	2.43	1.55	3.82
**Sixtil (2)**	0.608	0.239	6.47	1	0.011	1.84	1.15	2.93
**Sixtil (3)**	0.244	0.252	0.94	1	0.334	1.28	0.78	0.78
**Sixtil (4)**	0.065	0.261	0.06	1	0.802	1.07	0.64	1.78
**Sixtil (5)**	−0.763	0.323	5.56	1	0.018	0.47	0.25	0.88
**Constant**	−2.418	0.187	166.45	1	<0.0001	0.09		
**b]**	**B**	**S.E.**	**Wald**	**df**	**Sig.**	**Exp(B)**	**95% C.I.for EXP(B)**	
							**Lower**	**Upper**
Groups			28.172	5	<0.0001			
Sixtil (1)	0.726	0.247	8.598	1	0.003	2.066	1.272	3.356
Sixtil (2)	0.525	0.250	4.405	1	0.036	1.690	1.035	2.758
Sixtil (3)	0.140	0.263	0.285	1	0.594	1.151	0.687	1.926
Sixtil (4)	−0.058	0.277	0.044	1	0.834	0.944	0.549	1.623
Sixtil (5)	−0.679	0.330	4.247	1	0.039	0.507	0.266	0.967
Pre Systolic Blood Press	0.007	0.004	3.778	1	0.052	1.007	1.000	1.014
Pre Diastolic Blood Press	0.003	0.004	0.375	1	0.541	1.003	0.994	1.011
Albumine	−0.428	0.167	6.545	1	0.011	0.652	0.469	0.905
Ultrafiltration	0.0001	0.0001	12.316	1	<0.0001	1.000	1.000	1.000
Effective Dialysis Time	0.0001	0.004	0.005	1	0.945	1.000	0.992	1.008
Delta Temperature	0.023	0.107	0.048	1	0.827	1.024	0.830	1.262
Post Weight	−0.009	0.006	2.197	1	0.138	0.991	0.980	1.003
Constant	−2.214	1.300	2.903	1	0.088	0.109		

Table 5a) shows the association between convective volume and the occurrence of IDH without adjustment, and b) shows the Odds ratio after adjustment for relevant parameters.

The groups of convective volume were defined as follows: Group 1: 0 to 7.53 liters, Group 2: 7.54 to 14.8 liters; Group 3: 14.9 to 16.96 liters; Group 4: 16.97 to 18.9 liters; Group 5: 19.1 to 21 liters, and Group 6: 21.1 to 30 liters.

**Table 6:** Logistic regression analysis to determine Odds Ratio of hemodiafiltration volume for Intradialytic Hypotension

	**B**	**S.E.**	**Wald**	**Sig.**	**Exp[B]**	**95,0% C.I. for EXP[B]**	
						**Lower**	**Upper**
Nhdfvol			43.328	<0.0001			
Nhdfvol (1)	−0.115	0.760	0.023	0.879	0.891	0.201	3.952
Nhdfvol (2)	0.880	0.236	13.976	<0.0001	2.412	1.520	3.827
Nhdfvol (3)	0.600	0.244	6.045	0.014	1.822	1.129	2.939
Nhdfvol (4)	0.236	0.257	0.843	0.358	1.266	0.0765	2.096
Nhdfvol(5)	0.058	0.266	0.047	0.829	1.059	0.629	1.784
Nhdfvol (6)	−0.770	0.327	5.547	0.019	0.463	0.244	0.879
Constant	−2.410	0.194	154.604	<0.0001	0.090		

The groups of convective volume were defined as follows: Group 1: 0 to 0.2 liters. Group 2: 0.3 to 7.53 liters, Group 3: 7.54 to 14.8 liters; Group 4: 14.9 to 16.96 liters; Group 5: 16.97 to 18.9 liters; Group 6: 19.1 to 21 liters, and Group 7: 21.1 to 30 liters.

**Table 7:** Ultrafiltration rate by time

	**Groups**							**Dunett Test**		
	**1**	**2**	**3**	**4**	**5**	**6**	**Total**	**Anova**	**P < 0.05**	**P > 0.05**
UF rate 15 min (mL/min)	20 ± 14	23 ± 17	22 ± 17	26 ± 24	23 ± 20	23 ± 22	23 ± 19	<0.001	**1** vs. 4.	**1** vs 2,3,5,6
UF rate 30 min (mL/min)	20 ± 8	23 ± 9	21 ± 9	22 ± 9	20 ± 8	17 ± 8	20 ± 9	<0.0001	**1** vs 2–4.	**1** vs 5,6.
UF rate 45 min (mL/min)	18 ± 8	21 ± 8	19 ± 8	20 ± 9	18 ± 7	16 ± 8	19 ± 8	<0.0001	**1** vs 2,4.	**1** vs 3,5,6.
UF rate 60 min (mL/min)	16 ± 6	18 ± 8	17 ± 8	18 ± 7	17 ± 9	15 ± 6	17 ± 7	<0.0001	**1** vs 2–4.	**1** vs 5,6.
UF rate 75 min (mL/min)	14 ± 6	17 ± 6	15 ± 6	16 ± 6	15 ± 6	13 ± 6	15 ± 6	<0.0001	**1** vs 2–4.	**1** vs 5,6.
UF rate 90 min (mL/min)	12 ± 5	15 ± 6	14 ± 6	14 ± 5	13 ± 5	12 ± 5	13 ± 5	<0.0001	**1** vs 2–5.	**1** vs 6.
UF rate 105 min (mL/min)	11 ± 6	13 ± 6	13 ± 5	12 ± 5	12 ± 5	11 ± 4	12 ± 5	<0.0001	**1** vs 2–4.	**1** vs 5,6.
UF rate 120 min (mL/min)	10 ± 6	13 ± 6	12 ± 5	11 ± 5	11 ± 5	10 ± 4	11 ± 5	<0.0001	**1** vs 2–4.	**1** vs 5,6.
UF rate 135 min (mL/min)	10 ± 5	12 ± 5	11 ± 5	11 ± 5	10 ± 4	9 ± 4	10 ± 5	<0.0001	**1** vs 2–4.	**1** vs 5,6.
UF rate 150 min (mL/min)	9 ± 6	11 ± 6	10 ± 5	10 ± 5	9 ± 4	8 ± 4	9 ± 5	<0.0001	**1** vs 2–4.	**1** vs 5,6.
UR rate 165 min (mL/min)	8 ± 5	10 ± 5	9 ± 5	9 ± 5	8 ± 4	7 ± 4	9 ± 5	<0.0001	**1** vs 2–4.	**1** vs 5,6.
UF rate 180 min (mL/min)	8 ± 9	11 ± 9	10 ± 9	10 ± 10	8 ± 7	7 ± 5	9 ± 8	<0.0001	**1** vs 2–4.	**1** vs 5,6.
UF rate 210 min (mL/min)	9 ± 22	15 ± 22	11 ± 14	10 ± 9	10 ± 25	7 ± 8	10 ± 16	<0.0001	**1** vs 2.	**1** vs 3–6.

The groups of convective volume were defined as follows: Group 1: 0 to 0.2 liters. Group 2: 0.3 to 7.53 liters, Group 3: 7.54 to 14.8 liters; Group 4: 14.9 to 16.96 liters; Group 5: 16.97 to 18.9 liters; Group 6: 19.1 to 21 liters, and Group 7: 21.1 to 30 liters.

## Discussion

### Statement of principal findings

The present study shows that the group with the highest convective volume used for HDF has the lowest occurrence of IDH. This association was independent of relevant clinical factors such as pre HDF SBP, albumin, ultrafiltration rate, pre HDF body temperature and post HDF weight. A confirmatory analysis where patients receiving treatment with no convective volume were used as the reference group showed results consistent with the primary analysis. A non-significant trend of increasing risk of IDH with decreasing convective volume may be interpreted in Table 5a and b.

### Strengths

It may be outlined that potential confounders such as dialysate temperature, dialysate sodium and calcium did not significantly differ between the groups, which may be considered a strength of the present analysis. It is also important to note that potential confounders of the analysis such as ultrafiltration rate and albumin, which are main determinants of vascular refilling in response to interdialytic fluid removal, were included in an adjusted Logistic Regression model and did not alter the results. Particularly the inclusion of ultrafiltration rate in the analysis reduces the possible confounding influence caused by the significant differences found in interdialytic weight gain (Table 1). Inclusion of pre HDF SBP excludes the possibility that patients with higher SBP may have larger decreases (and thus higher likelihood of experiencing IDH) or those with lower SBP may have lower cardiac function and thus more likely to experience IDH. An additional strength of this analysis is the sample size which is considerable large with 2276 HDF treatments in 154 included subjects.

### Weaknesses of the study

Limitations of the current study are the retrospective nature of the analysis it is based on a re analysis of available data, and the considerably homogenous study population recruited in an urban dialysis facility in Mexico City. This trial has no information about water transfer from the vascular into the interstitial space, effective circulating volume, accurate information on mass transfer of solutes possibly relevant for hemodynamic stability (in particular sodium or calcium) and measurement of inflammatory markers serum interleukins. Additional confounding may have occurred due to differences in change of body core temperature which may alter the vascular response to removal of intravascular volume. No measurements of body core temperature were performed. Peripheral pre and post temperature was measured by blood thermal monitor and did not show significant differences (Table 1).

### Strengths and weaknesses in relation to other studies

Very few of the studies comparing HD and HDF have taken into account that these techniques differ in the operative settings of several variables potentially affecting the hemodynamic response to the procedure, namely, the rate of convective transport, the efficiency of small and large solute removal, the buffer used in the dialysate, sodium balance, treatment time and the type of membrane. A recently reported study in 34 patients reported that replacement volumes greater than 16 liters HDF not have a protective effect of hypotension [20]. Is this trial Pinney, et al. used a replacement volume from 16 to 21 liters. In general the results are consistent with our results because the 16-liter volume used in our work gives an OR of 1.059 (0.629 to 1.784). As can be seen in Figure 1, the convective volumes greater than 21 liters are those which decrease the risk of IDH. Several studies, carried out subsequently, reinforced the belief that procedures based on convective transport are superior to those based on diffusive transport in protecting the stability of blood pressure and heart rate [2-4,6,7].

### Possible mechanisms

Strong support in favor of HDF came from a series of hemodynamic studies showing that, for equal amounts of fluid removed, HDF elicited an appropriate increase in peripheral vascular resistance, whereas standard HD failed to do so [8]. Shaldon et al. hypothesized that the dialysate itself must be “the” vascular stabilizer with a rationale interleukin removal as the framework of the hypothesis [21]. Interleukins increase capillary permeability, resulting in water capillary leakage from the vascular into the interstitial space. This hypothesis has not yet specifically been investigated for HDF treatments, but recently a reduction of inflammatory markers in patients receiving HDF treatments has been reported [12]. To what extent this is related and consistent with the hypothesis of Shaldon et al. requires further research. An additional aspect which also requires more research is the mass transfer of sodium and calcium. Although a positive sodium transfer will raise the osmolality and quite likely result in increased interdialytic weight gains and blood pressure, a negative sequelae which should be avoided in patients suffering from renal impairment. However, it may not be excluded that mast transfer of sodium confounded our results by changing serum osmolality and affecting hemodynamic stability. The same accounts for calcium mass transfer, which is also of hemodynamic importance for patients receiving renal replacement therapy according to work by van der Sande et al. [22]. The techniques that are based on convective transport also entail a significant cooling of the blood flowing in the extracorporeal circuit [23-27] which may also affect hemodynamic stability. This is because in an isothermal treatment, difference between arterial line temperature (from the patient) and the venous line temperature (from the machine) is greater than 0.5 degrees Celsius for the arterial line (measured by Blood Thermal Monitor).

## Conclusions

At this point no definitive statement can be given and additional research is needed to confirm the findings of this research in a prospective setting. However, in summary the data of this retrospective cohort study shows an inverse correlation between the occurrence of intradialytic hypotension and convective volume prescription. This study is limited by its retrospective design and, although quite promising initial results in terms of hemodynamic stability are shown, much additional research is needed.

## Competing interests

All authors have no competing interests.

## Authors’ contributions

FMB participated in the design of the study, acquired the data, managed the data analysis and interpretation of the data, and drafted the manuscript. JGR substantially contributed to the interpretation of the data and critically revised the manuscript. GDC participated in the design of the study, acquired the data, and managed the data. SR assisted in the study design, performed the statistical analysis, contributed to the interpretation of the data, and critically revised the manuscript. AM participated in the study design and data interpretation, and critically revised the manuscript. HPG conceived of the study, participated in the study design and critically revised the manuscript. All authors read and approved the final manuscript.

## Pre-publication history

The pre-publication history for this paper can be accessed here:

http://www.biomedcentral.com/1471-2369/13/106/prepub
